# Geochemical characteristics and detrital zircon U-Pb ages of the Yimin Formation, Kelulun Depression, Hailar Basin and constraints on uranium mineralization

**DOI:** 10.1371/journal.pone.0309433

**Published:** 2024-08-30

**Authors:** Fanmin Meng, Fengjun Nie, Fei Xia, Zhaobin Yan, Da Sun, Wenbo Zhou, Xin Zhang, Qing Wang

**Affiliations:** 1 School of Earth Sciences, East China University of Technology, Nanchang, Jiangxi Province, China; 2 State Key Laboratory of Nuclear Resources and Environment, East China University of Technology, Nanchang, Jiangxi Province, China; 3 Geological Party No. 243, CNNC, Chifeng, Inner Mongolia, China; Hefei University of Technology School of Resources and Environmental Engineering, CHINA

## Abstract

The sandstone uranium deposits in the Kelulun Depression are the first commercially viable uranium deposits discovered in the Hailar Basin and the ore-bearing strata corresponding to the Lower Cretaceous Yimin Formation. However, the source of sedimentary matter, uranium source conditions, and uranium mineralization processes in the region have not been characterized. Accordingly, we analyzed the lithology, whole-rock geochemistry, zircon U-Pb ages, and trace elements of the Yimin Formation sandstones. The Yimin Formation sandstones were primarily composed of detrital grains with low compositional maturity. A geochemical analysis indicated that the parent rocks are felsic igneous rocks formed at an active continental margin with a moderately high degree of weathering. The detrital zircon U-Pb ages of the Yimin Formation 215–287 Ma with a peak at 230–260 Ma. Based on chronological, geochemical, and lithological data, we conclude that the Yimin Formation matter is derived from the Adunchulu Uplift on the western side of the Kelulun Depression and its parent rocks are Triassic granites. The Adunchulu uplift since the late Early Cretaceous and weathering and denudation of its uranium-rich granites provided ample matter and uranium. Therefore, the Kelulun Depression is a promising area for the exploration of sandstone uranium deposits.

## 1. Introduction

Sandstone uranium deposits are often very large, environmentally friendly to mine, and have low burial depths and mining costs. As such, they have become the primary focus of uranium exploration efforts across the world [1–3]. Large and ultra-large sandstone uranium deposits have been discovered in the Yili, Junggar, Ordos, Songliao, and Erlian Basins of China [4–11]. Recently, sandstone-related uranium deposits of considerable size have also been discovered in the Cretaceous strata of the Hailar Basin in northeastern Inner Mongolia.

The Hailar Basin is rich in hydrocarbon resources, such as coal, petroleum, and natural gas [12]. Based on sandbody characteristics [13,14], hydrogeological conditions [15], airborne radiometric survey data [16], and prospecting analyses [17–19], the geological conditions of the Hailar Basin are conducive to uranium mineralization. Many uranium points and deposits have recently been discovered in the Lower Cretaceous Yimin Formation of the Kelulun Depression, in the southwestern part of the Hailar Basin. Meng (2024) used electron microprobe to reveal the types and occurrence states of uranium minerals in the Yimin Formation, and elemental geochemical characteristics revealed the rock types, tectonic setting, sedimentary environment and paleoclimatic conditions in the source area of the Yimin Formation [20]. However, the geochemical data can only be used to determine the parent rock type in the source area of the Yimin Formation, but cannot accurately pinpoint the provenance. The research level of the Yimin Formation strata in the area is still relatively low, especially the source of the clastic material, sedimentary system, uranium source conditions, and uranium mineralization mechanisms, limiting the exploration of sandstone-related uranium deposits in this area. To address this problem, we analyzed the lithology, geochemistry, and zircon U-Pb ages of samples from the Lower Cretaceous Yimin Formation in the Kelulun Depression to reveal the sources of detrital matter, lithology of the parent rock, and tectonic setting and uranium fertility of the source region. Uranium mineralization processes were then inferred from the tectonic evolution of the source region and the aforementioned analyses. The study results are expected to guide future explorations for sandstone uranium deposits in the Hailar Basin.

## 2. Geological background

**Fig 1:**
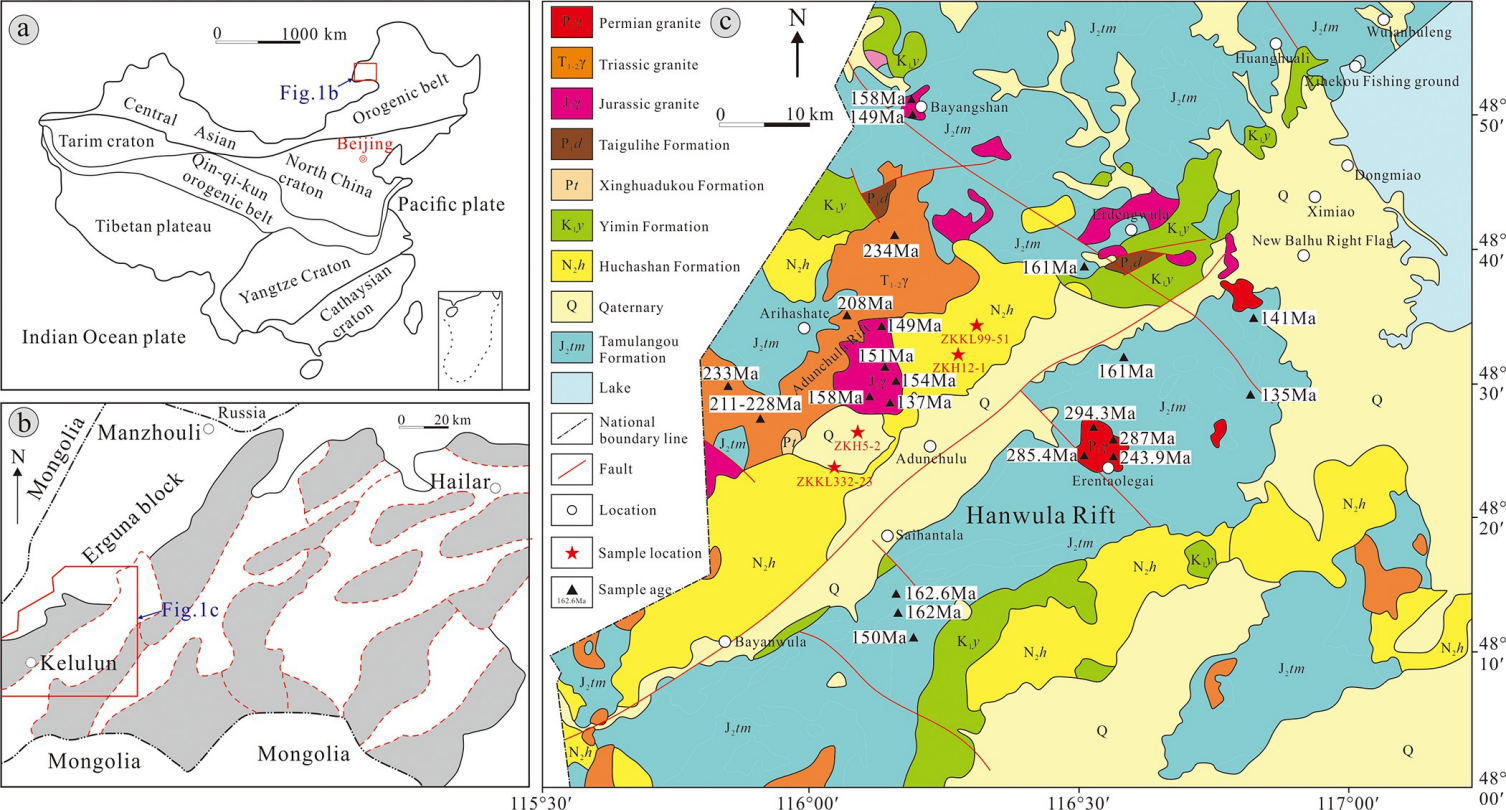
Geological map of the study area [20]. a. Geotectonic map of the Hailar Basin. b. Tectonic subdivisions of the Hailar Basin. c. Geological map of the Kelulun Depression. Republished from [Meng F.M, Nie F.J, Xia F, Yan Z.B, Sun, D, Zhou W.B, et al. Sedimentary Environment, Tectonic Setting, and Uranium Mineralization Implications of the Yimin Formation, Kelulun Depression, Hailar Basin, China. 2024; 12, 763.] under a CC BY license, with permission from [Journal of Marine Science and Engineering], original copyright [2024].

The Hailar Basin, located in the eastern Central Asian Orogenic Belt (Fig 1A), is a Mesozoic-Cenozoic continental rift basin that developed between the Siberian and North China Plates [21–23]. The basin is controlled by the NE-striking Deerbugan and Ergun fault zones, and it has undergone four stages of tectonic evolution, a rifting stage in the Late Jurassic, a syn-rifting stage during the Early Cretaceous, a shrinking stage in the late Early Cretaceous, and differential uplift and denudation during the Late Cretaceous-Neogene [24–29], which formed a tectonic pattern with “two uplifts and three depressions.” (Fig 1B) [30,31].

The Kelulun Depression is a long and narrow half-graben in the southwestern part of the Hailar Basin, which trends in the NE direction and is bounded by faults on its western side (Fig 1C). The basement of the basin is comprised of Paleozoic metamorphic rocks and Hercynian granites, with many granites and intermediate-to-acidic and intermediate-to-alkaline igneous rocks around the basin. In descending chronological order, its sedimentary strata are the Lower Cretaceous Nantun, Tongbomiao, Damoguaihe, and Yimin Formations, the Neogene Huchashan Formation, and Quaternary strata. The ore-bearing strata in the study area are the Lower Cretaceous Yimin Formation, consisting of dark coal-bearing clastic sediments with high reducing capacities. Uranium mineralization has been discovered in the fan deltas of the Yimin Formation.

## 3. Sample acquisition and analysis

Whole-rock geochemical analyses were performed on 24 sandstone samples from boreholes ZKH5-2, ZKH12-1, ZKKL332-23, and ZKKL99-51 in the Kelulun Depression, were obtained from gray-white sandstone in the Yimin Formation, via four boreholes. The sample locations are shown in Fig 1C. Preparation of the thin rock sections, the selection of zircons, target preparation, and cathodoluminescence (CL) microscopy were performed by the Langfang Geological Exploration Technology Service Co., Ltd.

### 3.1 Major and trace element analyses

Whole-rock geochemical analyses were performed at the Nuclear Geology and Nuclear Technology Application Center of Hunan Province. Major element analyses were performed using a Shimadzu XRF-1800 (Japan) X-ray fluorescence fluorometer, with an analytical accuracy of 5% or better. Trace and rare earth element analyses were performed via inductively coupled plasma mass spectrometry (ICP-MS), using a Thermo Fisher X Series II (USA) quadrupole mass spectrometer, which has analytical accuracies of <5% and <10% for trace element concentrations greater and less than 10 ppm, respectively.

### 3.2 U-Pb dating of detrital zircons

Zircon U-Pb dating was performed at the State Key Laboratory of Continental Dynamics at Northwest University, using a laser ablation-ICP-MS (LA-ICP-MS) system including a MicroLas GeoLas 200M laser ablation system and Agilent 7500a ICP-MS. A laser spot size of 35 μm was used during this process. The zircon U-Pb ages were determined by using the 91500 zircon standard as an external reference. The trace element compositions of the zircons were analyzed using NIST SRM 610 synthetic silicate glass and Si as external and internal standards [32], respectively. Data processing was performed using the ICP-MS-DataCal 9.2 software suite. The method of Andersen (2002) [33] was used for common Pb correction. The concordia and frequency plots of the zircon ages were created using Isoplot 4.15, the ages are used ^206^Pb/^238^U in interpretations.

## 4. Results

### 4.1 Lithological characteristics

**Fig 2:**
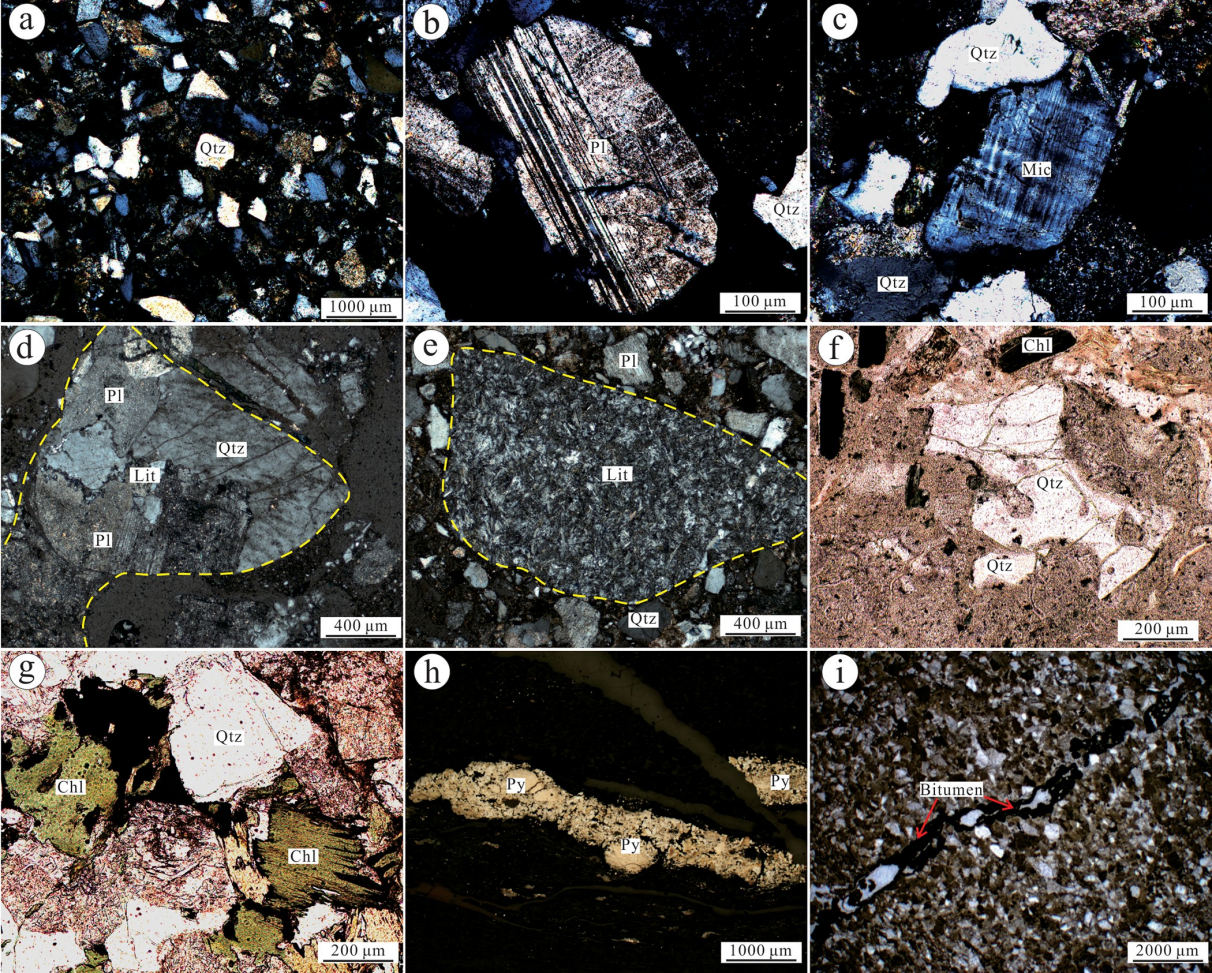
Photographs of thin sections under optical microscopy of the Yimin Formation sandstones. a. Medium-to-fine grained sandstones (cross-polarized). b. Twinned plagioclase crystal (cross-polarized). c. Grid-twinned microcline (cross-polarized). d. Granite fragment (cross-polarized). e. Andesite tuff (cross-polarized). f. Crystal tuff (plane-polarized). g. Chloritized biotite (plane-polarized). h. Pyrite vein (plane-polarized). i. Bitumen vein (plane-polarized).

The Yimin Formation sandstones are medium-to-fine grained (Fig 2A). Pore and contact cementation are dominant, with some parts showing calcareous cementation. The detrital grains are poorly sorted and rounded. Compositional maturity is low, indicative of short sediment transport distances from the source. Detrital grains account for 85%–90% of the whole-rock composition, while filling materials account for the remaining 10%–15%. Quartz accounts for 30%–40% of the detrital matter. The grains are angular-subangular and range from 0.10 mm to 0.42 mm, a small number exhibit undulatory extinction. Feldspar (mainly plagioclase, Fig 2B), with small amounts of microcline (Fig 2C) and striated feldspar) accounts for 10%–25% of the detrital matter and presented as angular-subangular grains that range from 0.13 mm to 0.48 mm. Clasts (mainly granite fragments (Fig 2D) and small amounts of andesite (Fig 2E) and crystal tuffs (Fig 2F) account for 20%–50% of the detrital matter and present as angular-subangular grains that range from 0.18 mm to 0.16 mm. Some of these grains had sizes exceeding 1.00 cm. The biotites present as flakes with intense chloritization and account for 2%–4% of the detrital matter (Fig 2G). Some rocks were also interspersed with pyrite (Fig 2H) and bitumen veins (Fig 2I).

### 4.2 Geochemical characteristics

#### 4.2.1 Major element characteristics

The major element composition of the Yimin Formation sandstones is shown in S1 Table. The Yimin Formation sandstones have SiO_2_ contents of 65.88%–74.92%, Al_2_O_3_ contents of 11.87%–15.98%, Fe_2_O_3_^T^ contents of 1.45%–6.37%, CaO contents of 0.53%–4.54%, K_2_O contents of 2.65%–3.97%, and Na_2_O contents of 1.78%–3.67%.

#### 4.2.2 Trace and rare element characteristics

**Fig 3:**
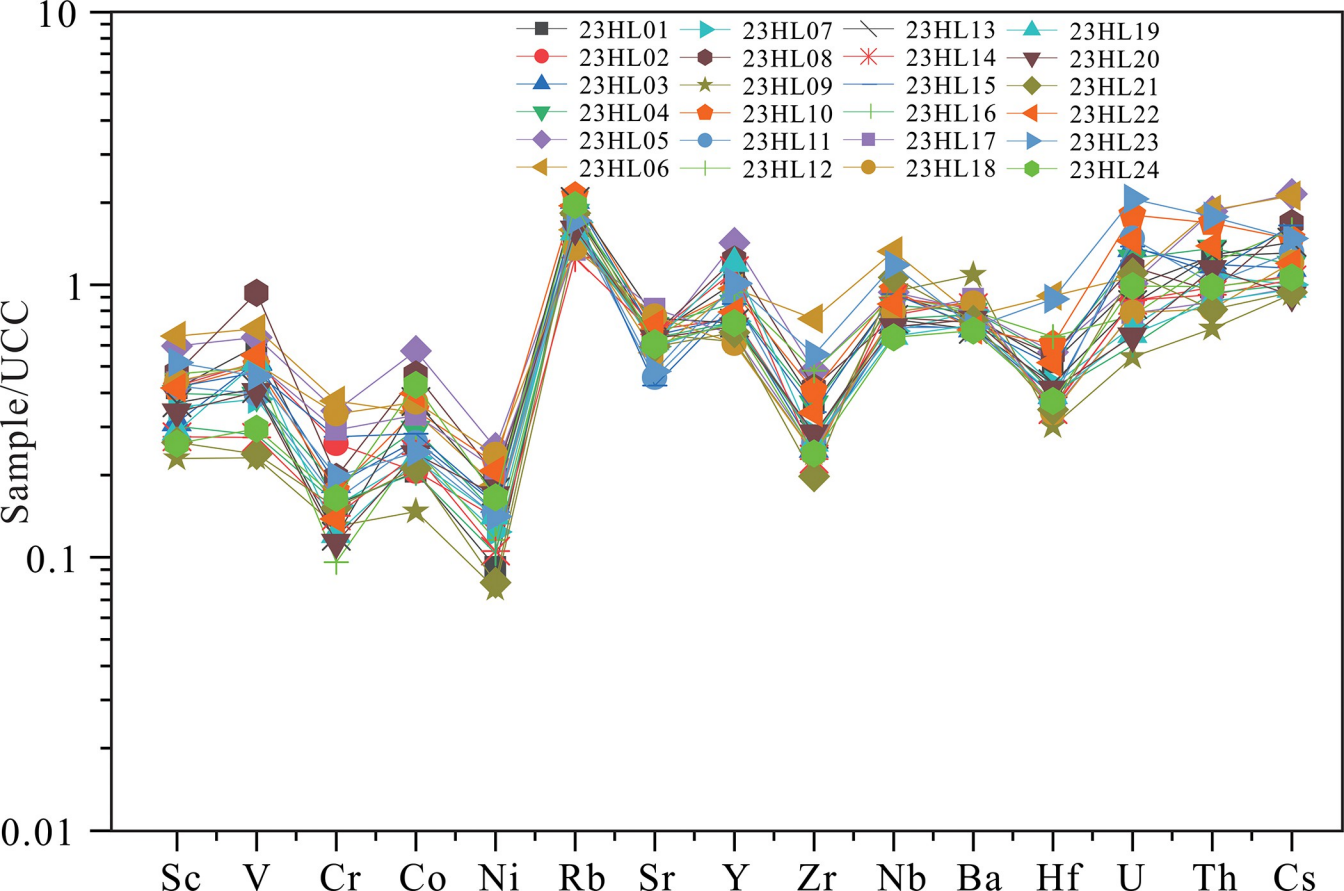
Upper continental crust-normalized trace element spidergram for samples from the Yimin Formation [34].

The trace and rare element compositions of the Yimin Formation sandstones are shown in S2 Table. The samples have similar trace element spidergram patterns, characterized by weak enrichment in V, Rb, Co, Y, Nb, and U and weak depletion in Sc, Cr, Ni, Sr, Zr, and Hf relative to concentrations in the upper continental crust (Fig 3).

**Fig 4:**
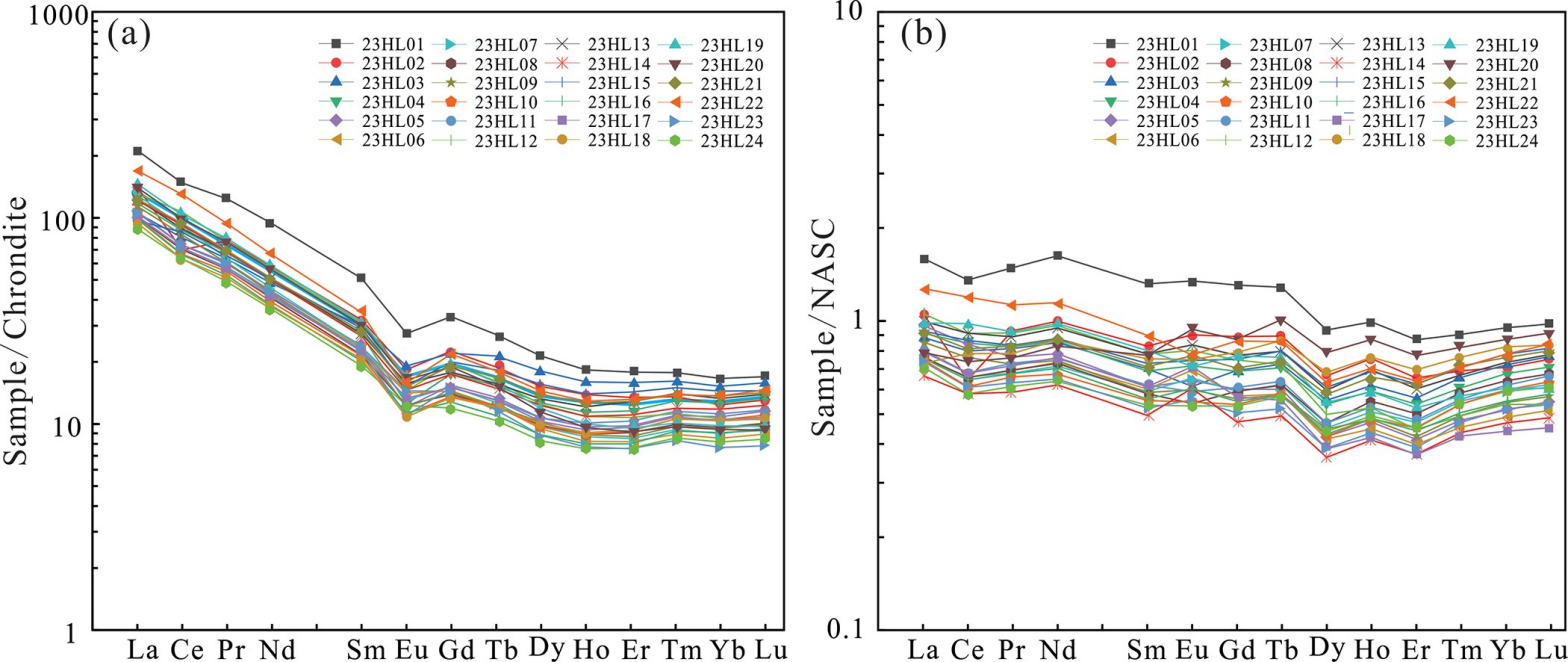
(a) Chondrite-normalized REE diagram [36]. (b) NASC-normalized REE diagram [37].

The total rare earth element (ΣREE), light rare earth element (LREE), and heavy rare earth element (HREE) contents of the samples are 93.1–226.3 ppm, 84.7–205.4 ppm, and 8.4–20.9 ppm, respectively, and LREE/HREE ratios range from 6.4 to 11.9 (S2 Table). Therefore, the samples show significant LEE/HREE fractionation, with LREE enrichment and HREE depletion. On the chondrite-normalized REE diagram (Fig 4A), these samples have a significant right-inclined pattern and negative Eu anomaly. On the North American shale composite (NASC)-normalized REE diagram, the curve is nearly flat (Fig 4B), suggesting that their REE composition resembles that of North American shales. Hence, the source rocks of the Yimin Formation are derived from the upper continental crust [35].

### 4.3 U-Pb dating of zircons

**Fig 5:**
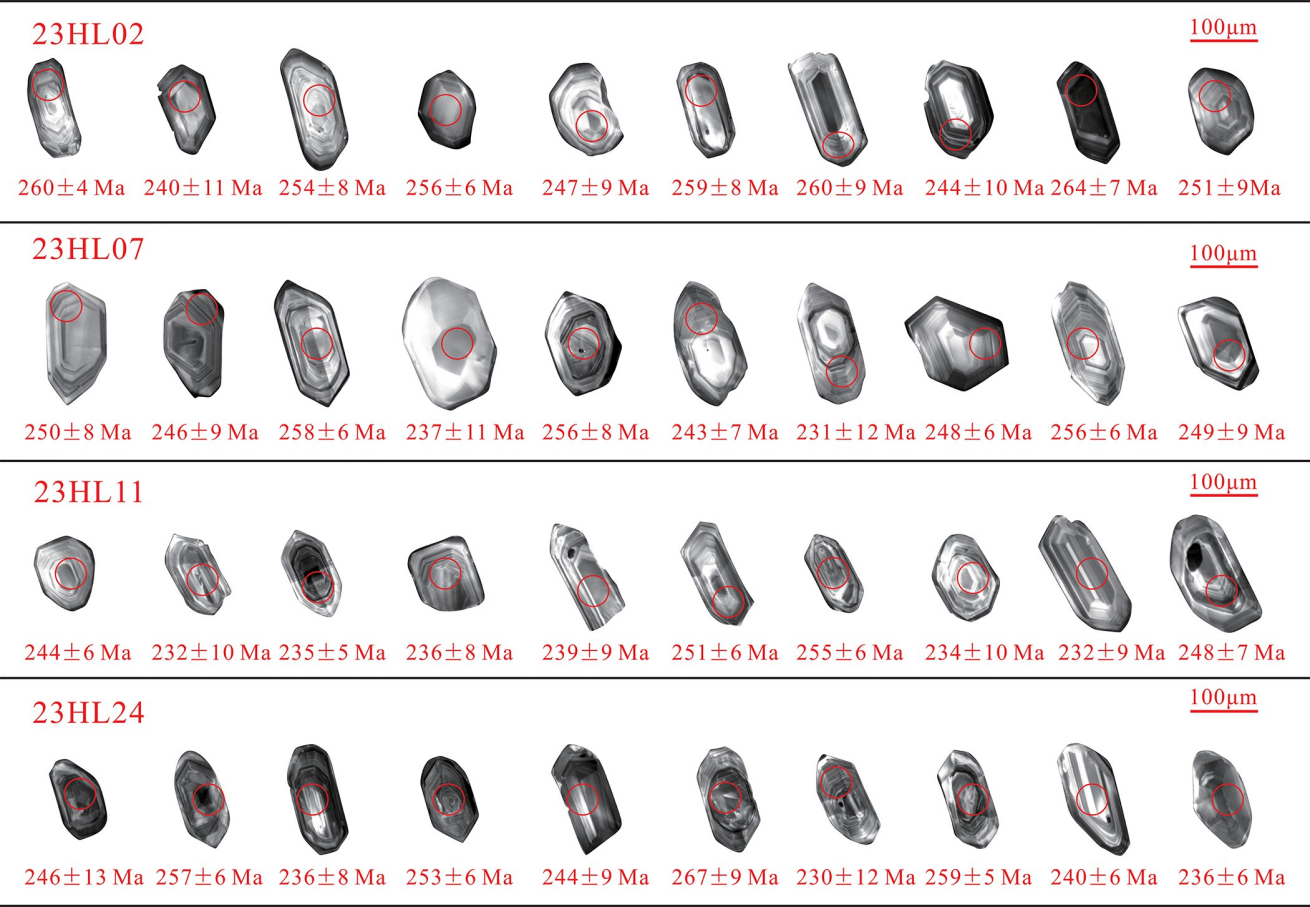
Cathodoluminescence images of detrital zircons from the Yimin Formation sandstones, with circles marking laser spot placement and number indicating the obtained ^206^Pb/^238^U ages.

**Fig 6:**
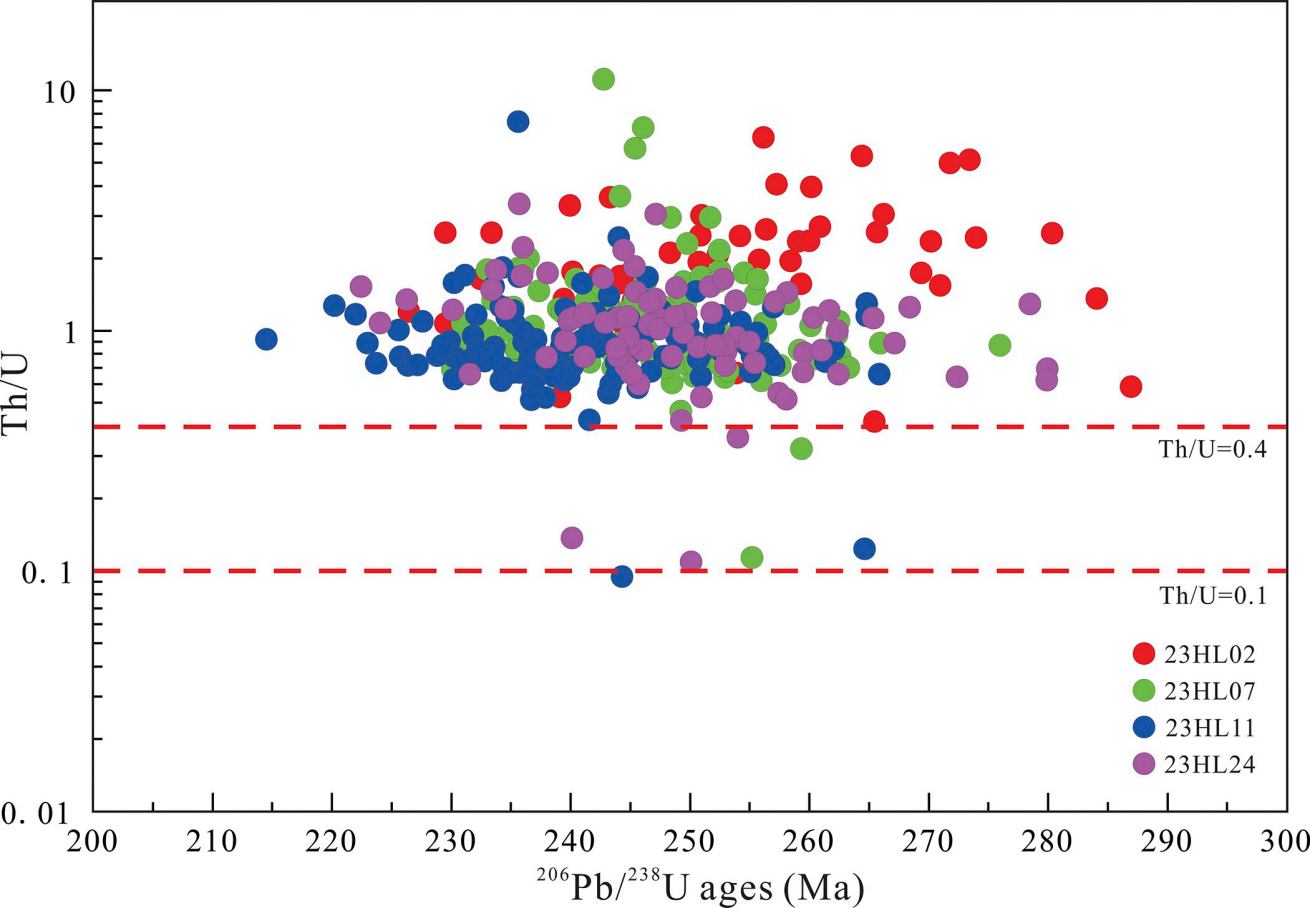
Relationship between zircon ages and Th/U in the Yimin Formation sandstones.

Zircons from the Yimin Formation present as euhedral-subhedral grains with long-axis diameters of 100–190 μm and aspect ratios of 1.1–1.8. Most of the grains are angular-subangular (Fig 5), consistent with short transport distances. As the majority of the zircon grains show oscillatory zoning, they are magmatic zircons. As shown in S2 Table, the Th/U ratios of the 23HL02, 23HL07, 23HL11, and 23HL24 samples are 0.4–6.3, 0.1–11.1, 0.1–7.4, and 0.1–3.4, respectively. Since the majority of these Th/U ratios are above 0.4 (Fig 6). Weathering of the parent rock increases Th/U, the Th/U ratios of 0.1–11.1 for detrital zircons, with most samples having values of 0.5–4.0, are consistent with substantial weathering of rocks in the source region [38,39].

**Fig 7:**
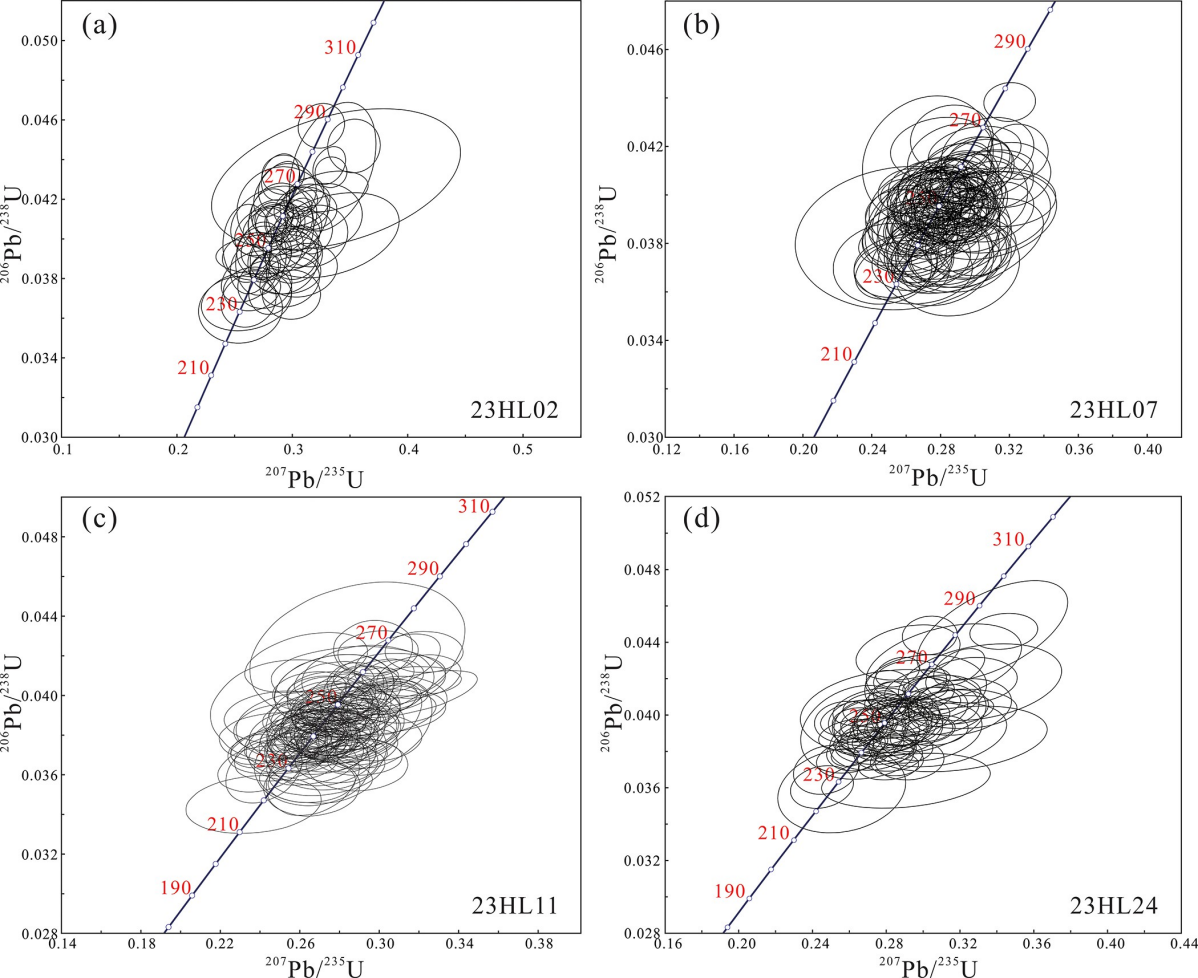
Concordia diagram of detrital zircon LA-ICP-MS U-Pb ages.

**Fig 8:**
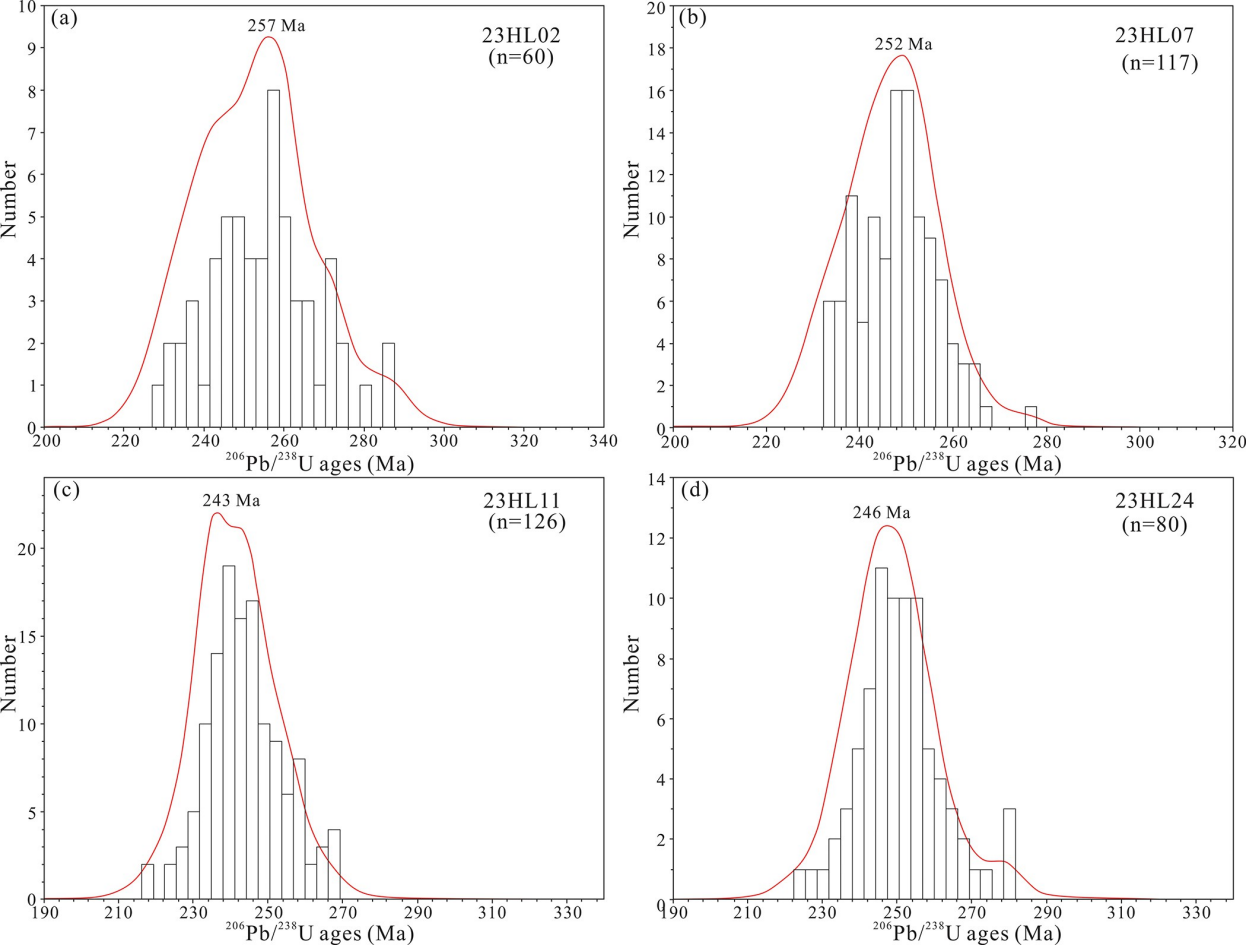
Age spectrum of detrital zircons from the Yimin Formation sandstones.

After discarding data with concordance <90%, 383 valid age data points were retained for geochronological analyses (S3 Table). In the age concordia diagram (Fig 7) and age spectrum (Fig 8), all four samples plot near the U-Pb concordia line, indicating that the age distributions are consistent with each other (concentrated in the 230–260 Ma range). In particular, the ^206^Pb/^238^U ages of zircons in the 23HL02, 23HL07, 23HL11, and 23HL24 samples are 230–270, 235–265, 230–260, and 235–260 Ma, respectively.

### 4.4 Trace element composition of the zircons

**Fig 9:**
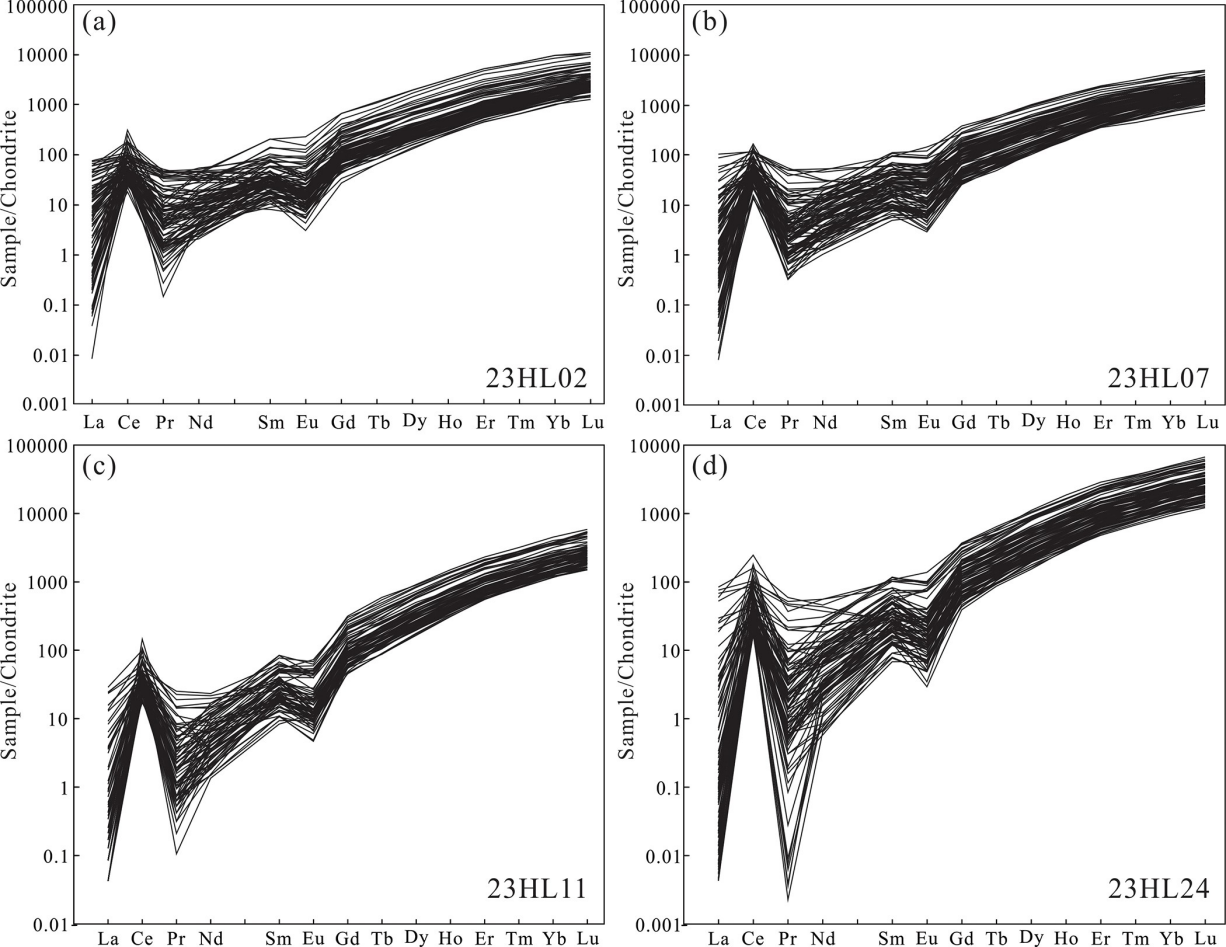
Chondrite-normalized REE fractionation curves of detrital zircons from the Yimin Formation sandstones [36].

The trace element characteristics of detrital zircons can be used to infer the types of rocks in the source region, the mechanism of zircon formation, and the tectonic setting of the source region [39]. As shown in S4 Table, zircons in the 23HL02, 23HL07, 23HL11, and 23HL24 samples have REE contents of 391.6 ppm to 3998.5 ppm, 377.9 ppm to 2219.9 ppm, 367.8ppm to 3113.36 ppm, and 441.69 ppm to 3418.94 ppm and LREE/HREE ratios of 0.028–0.311, 0.023–0.22, 0.026–0.1, and 0.028–0.302, respectively. Therefore, all four samples show LREE depletion and HREE enrichment. After chondrite normalization (Fig 9), all four samples have similar left-inclined REE fractionation curves, variable negative Eu anomalies, and positive Ce anomalies. Therefore, the detrital zircons in these samples retain the characteristics of magmatic zircons [40].

**Fig 10:**
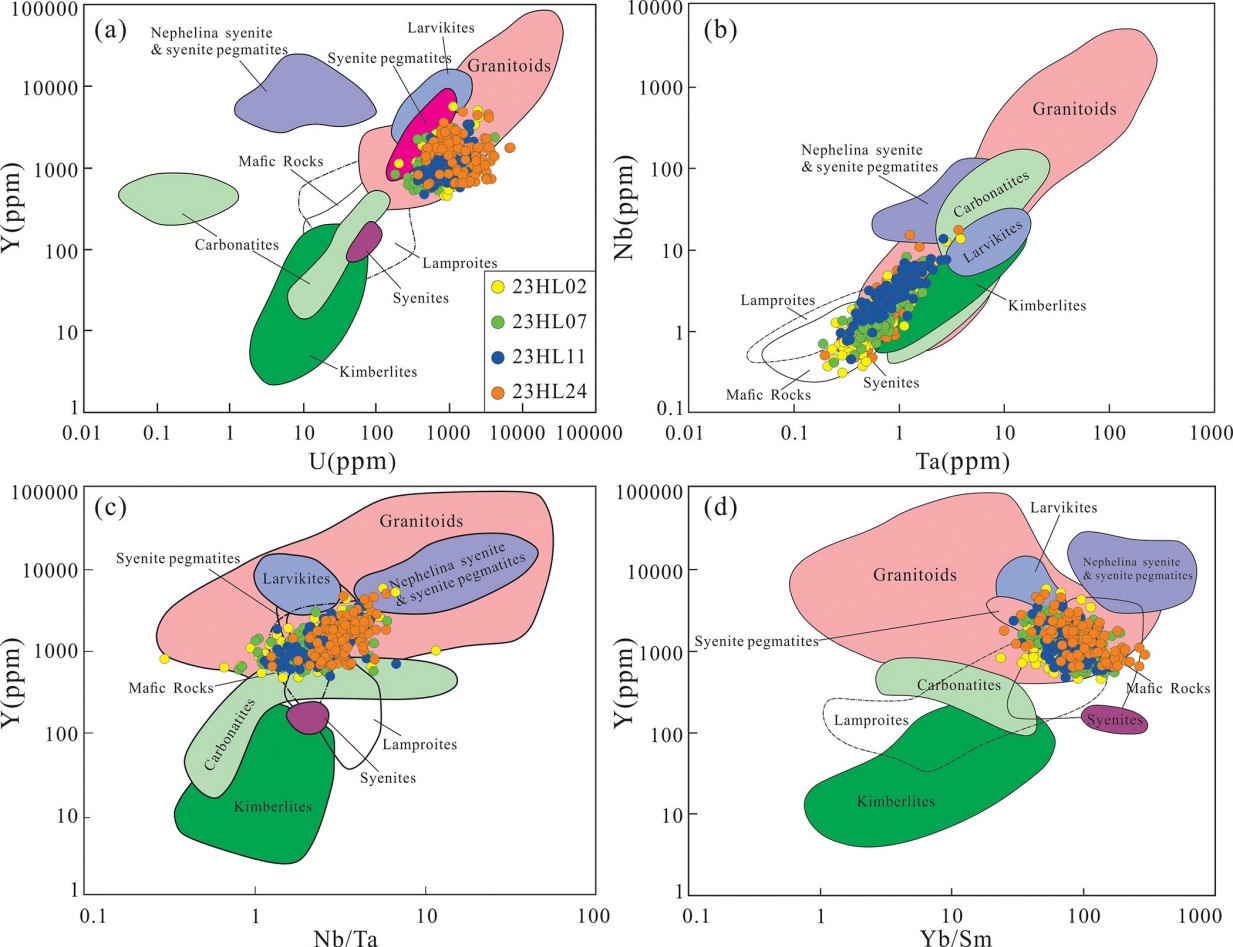
Zircon trace element discrimination diagrams for the Yimin Formation sandstones [41]. a. Y-U diagram. b. Nb-Ta diagram. c. Y-Nb/Ta diagram. d. Y-Yb/Sm diagram.

In the Y-U (Fig 10A) and Nb-Ta (Fig 10B) diagrams, the samples plot in the field of granites. In the Y-Nb/Ta diagram (Fig 10C), the samples mostly plot in the field of overlap between granites, syenite pegmatites, with some mafic rocks and a small number in the field of carbonate rocks. In the Y-Yb/Sm diagram (Fig 10D), the samples mainly plot in the field of overlap between granites, syenite pegmatites, and mafic rocks. Therefore, the parent rocks of the Yimin Formation sandstones are mainly granitoids.

## 5. Discussion

Trace elements (including REEs) of fine-grained clastic rocks are relatively stable in the later diagenesis and weathering processes, thus making them reliable tools for tracing the provenances and depositional settings [42–45].

### 5.1 Type of parent rocks and tectonic setting of the source

#### 5.1.1 Type of parent rocks

**Fig 11:**
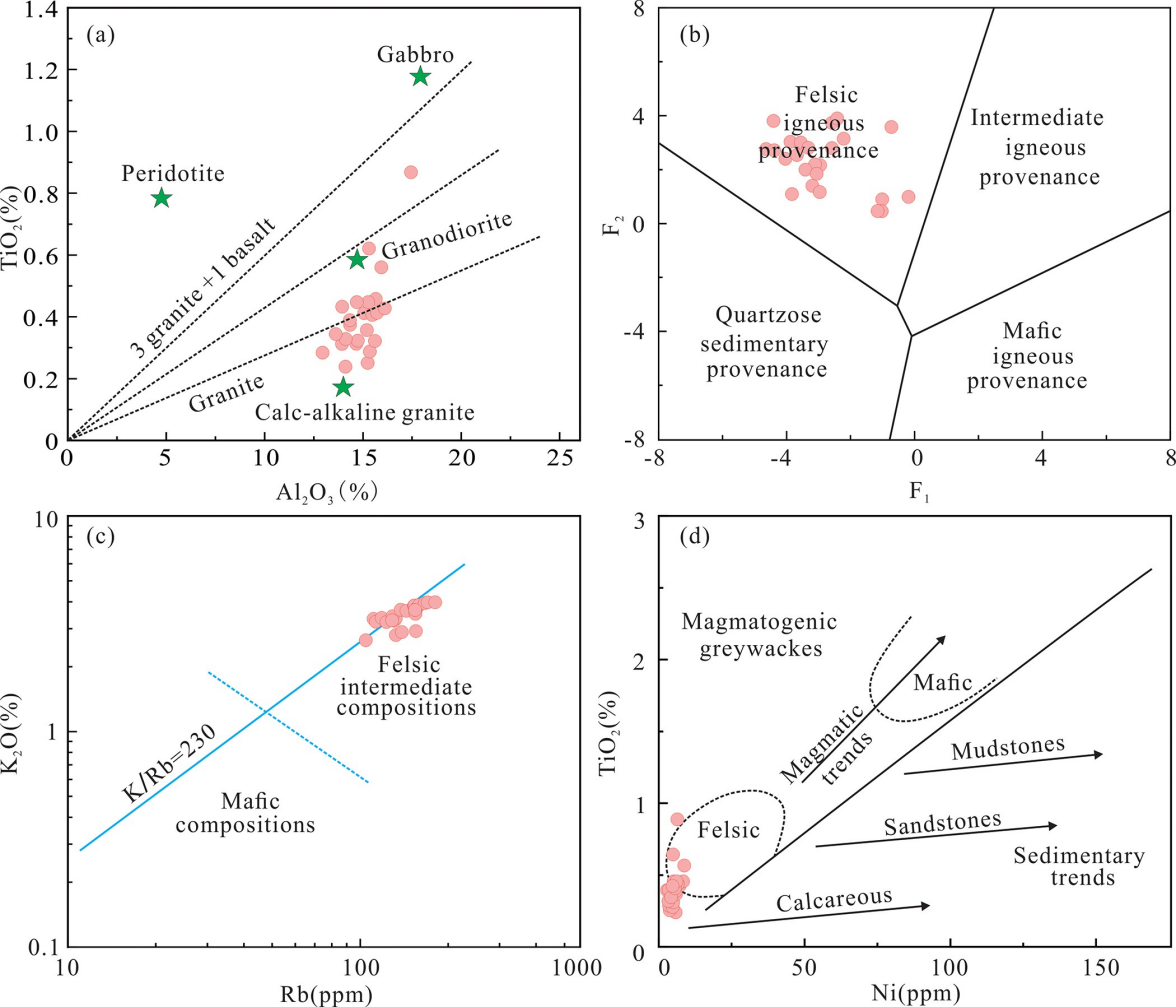
Major element discrimination diagrams for the parent rocks of the Yimin Formation sandstones. a. TiO_2_-Al_2_O_3_ diagram [47]. b. *F*_2_-*F*_1_ diagram [48]. c. K_2_O-Rb diagram [49]. d. TiO_2_-Ni diagram [50].

Girty (1996) showed that the Al_2_O_3_/TiO_2_ ratio is an effective parameter for discriminating the provenance of sandstones [46], where sandstones derived from mafic rocks have Al_2_O_3_/TiO_2_ ratios less than 8 and sandstones derived from intermediate and felsic igneous rocks have Al_2_O_3_/TiO_2_ ratios of 8–21 and >21, respectively. As the Yimin Formation sandstones have Al_2_O_3_/TiO_2_ ratios of 18.43–55.34, their parent rocks are felsic igneous rocks. In the TiO_2_-Al_2_O_3_ diagram (Fig 11A), the samples largely plot in the field of granites and granodiorites. In the *F*_2_-*F*_1_ diagram (Fig 11B), all of the sample plot in the field of felsic igneous rocks. In the K_2_O-Rb diagram (Fig 11C), they plot in the field of intermediate-to-acidic rocks. In the TiO_2_-Ni diagram (Fig 11D), they plot within and around the field of acidic rocks.

**Fig 12:**
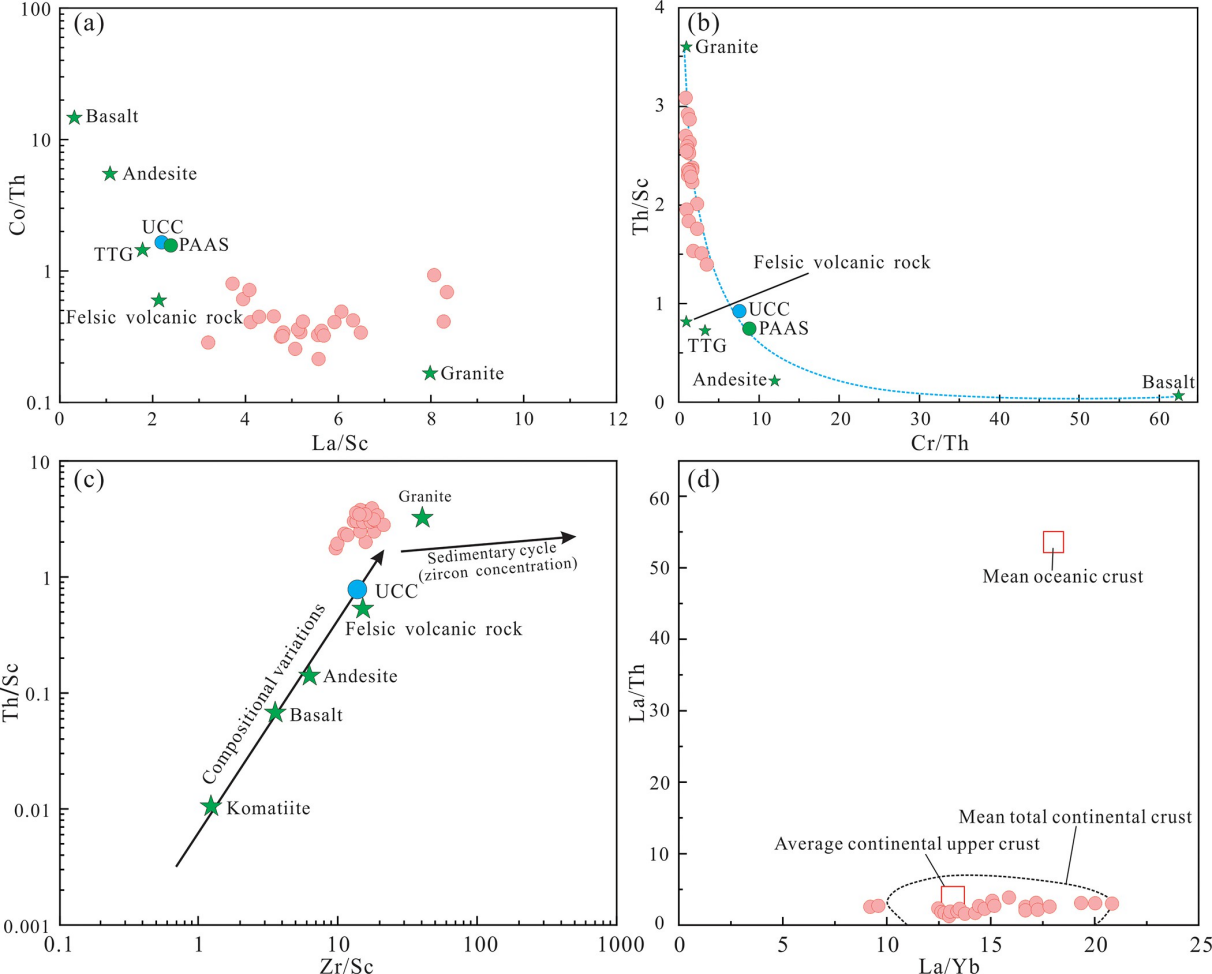
Trace element discrimination diagrams for the parent rocks of the Yimin Formation sandstones. a. Co/Th-La/Sc diagram [51]. b. Th/Sc- Cr/Th diagram [52]. c. Th/Sc-Zr/Sc diagram [53]. d. La/Th-La/Yb diagram [54]. UCC: Upper continental crust. PAAS: Post-archean Australian shale. TTG: Trondhjemite-tonalite-granodiorite.

**Fig 13:**
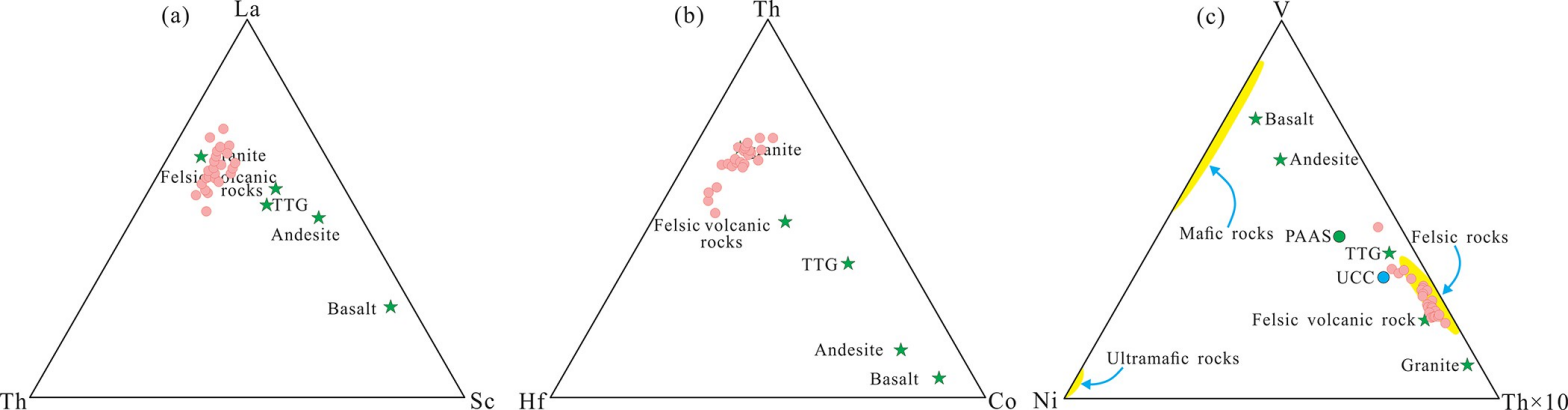
Triangular trace-element discrimination diagrams for the parent rocks of the Yimin Formation sandstones. a. La-Th-Sc diagram [55], b. Th-Hf-Co diagram [34], c. V-Ni-Th×10 diagram [52]. UCC: Upper continental crust. TTG: Trondhjemite-tonalite-granodiorite. PAAS: Post-archean Australian shale.

In the Co/Th-La/Sc diagram (Fig 12A), all samples plot in the field of felsic igneous rocks and granites. In the Th/Sc-Cr/Th (Fig 12B) and Th/Sc-Cr/Th diagrams (Fig 12C), they plot between felsic igneous rocks and granites. In the La/Th-La/Yb diagram (Fig 12D), all samples plot near the averages of the upper continental crust. In the La-Th-Sc (Fig 13A), Th-Hf-Co (Fig 13B), and V-Ni-Th×10 diagrams (Fig 13C), all the Yimin Formation samples plot in the field of granites and felsic igneous rocks. Therefore, the Yimin Formation sandstones were derived from felsic igneous rocks in the upper crust.

#### 5.1.2 Tectonic setting of the source region

**Fig 14:**
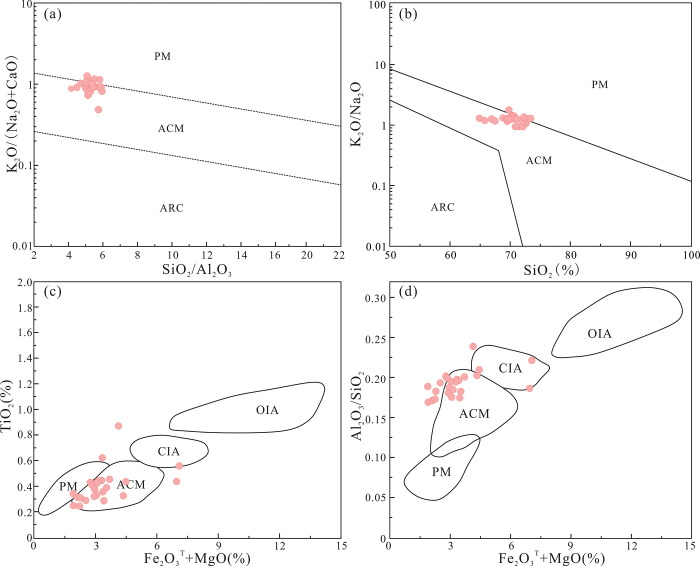
Major element discrimination diagrams for the tectonic setting of the Yimin Formation sandstones. a. K_2_O/(Na_2_O+CaO)-SiO_2_/Al_2_O_3_ diagram [56]. b. K_2_O/Na_2_O-SiO_2_ diagram [57]. c. TiO_2_-(Fe_2_O_3_^T^+MgO) diagram [58]. d. Al_2_O_3_/SiO_2_-(Fe_2_O_3_^T^+MgO) diagram [59]. PM: Passive continental margin. ACM: Active continental margin. ARC: Oceanic island arc margin. OIA: Oceanic island arc. CIA: Continental island arc.

**Fig 15:**
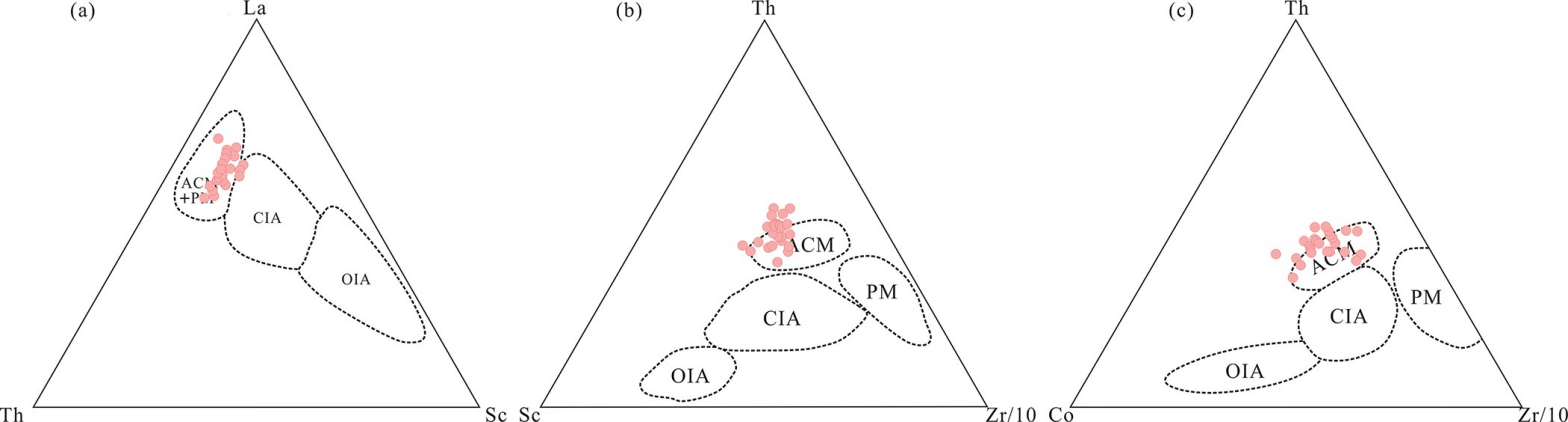
Trace element discrimination diagrams for the tectonic setting of the Yimin Formation sandstones. a. La-Th-Sc diagram, b. Th-Sc-Zr/10 diagram, c. Th-Co-Zr/10 diagram [58]. PM: Passive continental margin. ACM: Active continental margin. OIA: Oceanic island arc. CIA: Continental island arc.

Basin structures can be classified into one of four types according to their crustal characteristics: oceanic island arcs, continental island arcs, active continental margins (ACMs), and passive margins (PMs). The tectonic setting of a source region can be inferred using the presence of stable oxides like Al_2_O_3_, SiO_2_, TiO_2_, Fe_2_O_3_^T^, and MgO. In the K_2_O/(Na_2_O+CaO)-SiO_2_/Al_2_O_3_ (Fig 14A) and K_2_O/Na_2_O-SiO_2_ diagrams (Fig 14B), the samples from the Yimin Formation mostly plot in the field of ACMs. In the TiO_2_-(Fe_2_O_3_^T^+MgO) (Fig 14C) and Al_2_O_3_/SiO_2_-(Fe_2_O_3_^T^+MgO) diagrams (Fig 14D), these samples largely plot in the field of ACMs, with a few plotting outside but near this field. In the La-Th-Sc diagram (Fig 15A), all the Yimin Formation samples plot in the field of ACMs and PMs. In the Th-Sc-Zr/10 (Fig 15B) and Th-Sc-Zr/10 diagrams (Fig 15C), all of the samples plot in the field of ACMs.

The Hailar Basin was affected by the southward subduction of the Mongolia-Okhotsk Plate, resulting in extensive volcanic activity and the formation of a large number of Late Paleozoic granites in Manzhouli, southern Hailar Basin. The Late Paleozoic granites in the western part of the Kelulun Depression have undergone weathering, erosion, and transportation into the basin [60]. Because the composition and geochemical characteristics of clastic rocks are influenced mainly by the tectonic background of the source area, the complex tectonic background identified by the geochemical feature analysis does not represent the tectonic environment during the formation of detrital rocks but rather reveals the tectonic environment during the formation of the corresponding detrital source rocks. These findings suggest that the tectonic setting of the source region of the Yimin Formation sandstones was an ACM.

### 5.2 Paleoweathering characteristics of the source region

The chemical composition of a sandstone is influenced by weathering, transport, and sedimentation, as well as post-diagenetic processes. As weathering increases, sandstone shows the loss of mobile oxides, increase in stable oxides, and total element loss [61,62]. Therefore, the oxide content is an indicator of the degree of weathering of parent rocks in the source region [63,64].

**Fig 16:**
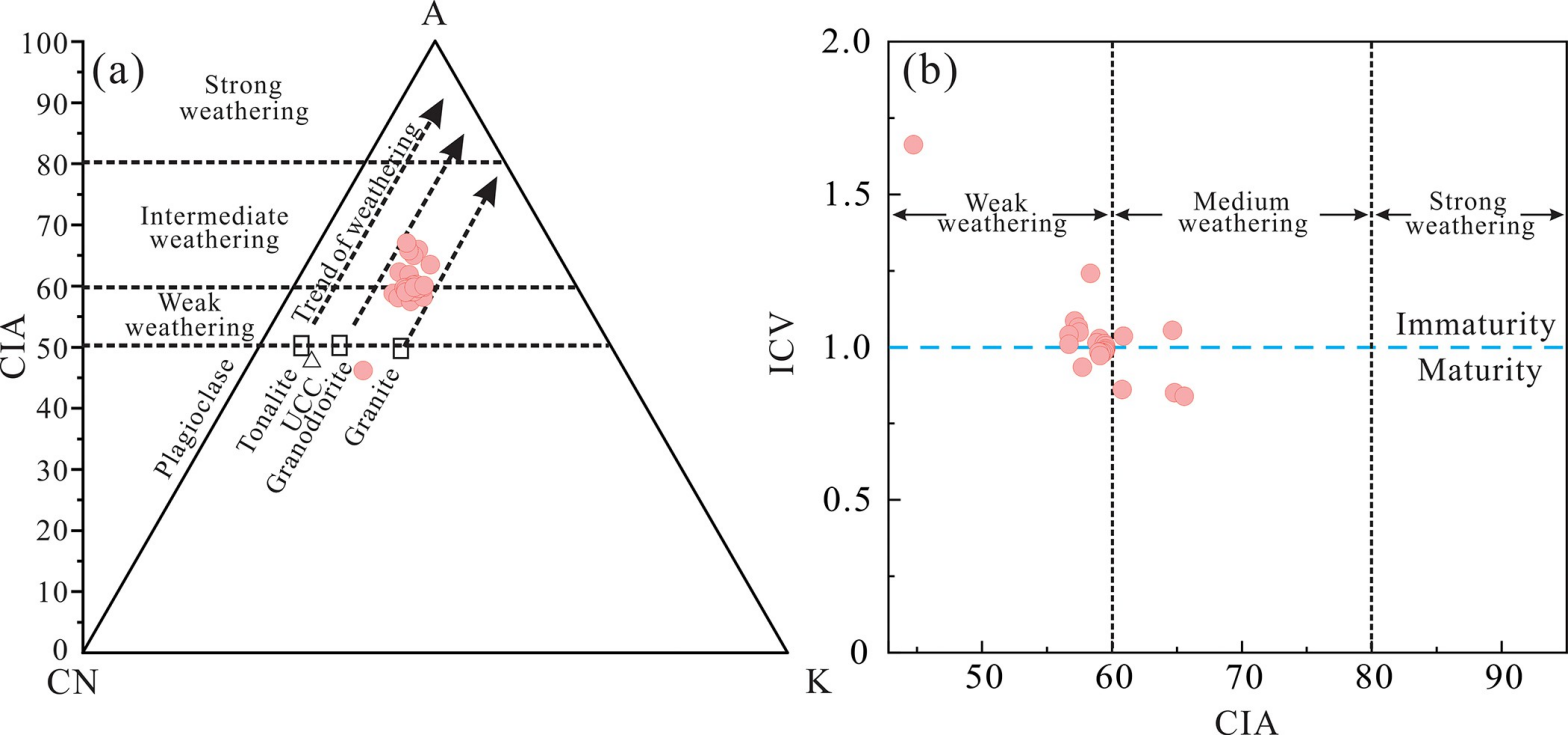
Discrimination diagrams for the paleoweathering characteristics of the source region of the Yimin Formation sandstones. a. A-CN-K diagram [70]. b. ICV-CIA diagram [63]. UCC: Upper continental crust.

Intense weathering of the parent rocks is supported by analyses of the Chemical Index of We-athering (CIW = 100Al_2_O_3_/(CaO*+Na_2_O+Al_2_O_3_), Chemical Index of Alteration (CIA = 100Al_2_O_3_/(Al_2_O_3_+CaO*+Na_2_O+K_2_O), and Index of Chemical Variability (ICV = (Fe_2_O_3_^T^+Na_2_O+K_2_O+CaO+MgO+TiO_2_)/Al_2_O_3_). Briefly, CIA values of 50–60, 60–80, and 80–100 are indicative of low, moderate, and intense chemical weathering [65], respectively. ICV < 1 and ICV > 1 indicate intense and relatively low weathering [66–68], respectively. CIW values of 50–60 indicate fresh rocks, and CIW > 70 indicates intense weathering [69]. The Yimin Formation sandstones have CIA values of 42.18–65.39 and CIW values of 48.50–76.26, indicating that their parent rocks were moderately to st-rongly weathered. Observed ICV values of 0.84–1.66 (mostly 0.85–1.0) indicate that the rocks in

the source region have been strongly weathered. In the A-CN-K diagram (Fig 16A), the CIA valu-es of the Yimin Formation sandstones are largely 55–70, indicating that the felsic rocks in their so-urce region have undergone moderate-to-strong weathering. The CIA-ICV diagram (Fig 16B) ind-icate that the Yimin Formation sandstones are compositionally immature, which is indicative of a moderately high degree of weathering in the parent rocks. In summary, the source region of the Yi-min Formation sandstones has undergone moderately strong weathering, promoting erosion of the parent rock and thus providing an ample supply of detrital matter for the study area.

### 5.3 Provenance analysis

#### 5.3.1 Constraints on provenance from regional sedimentology

**Fig 17:**
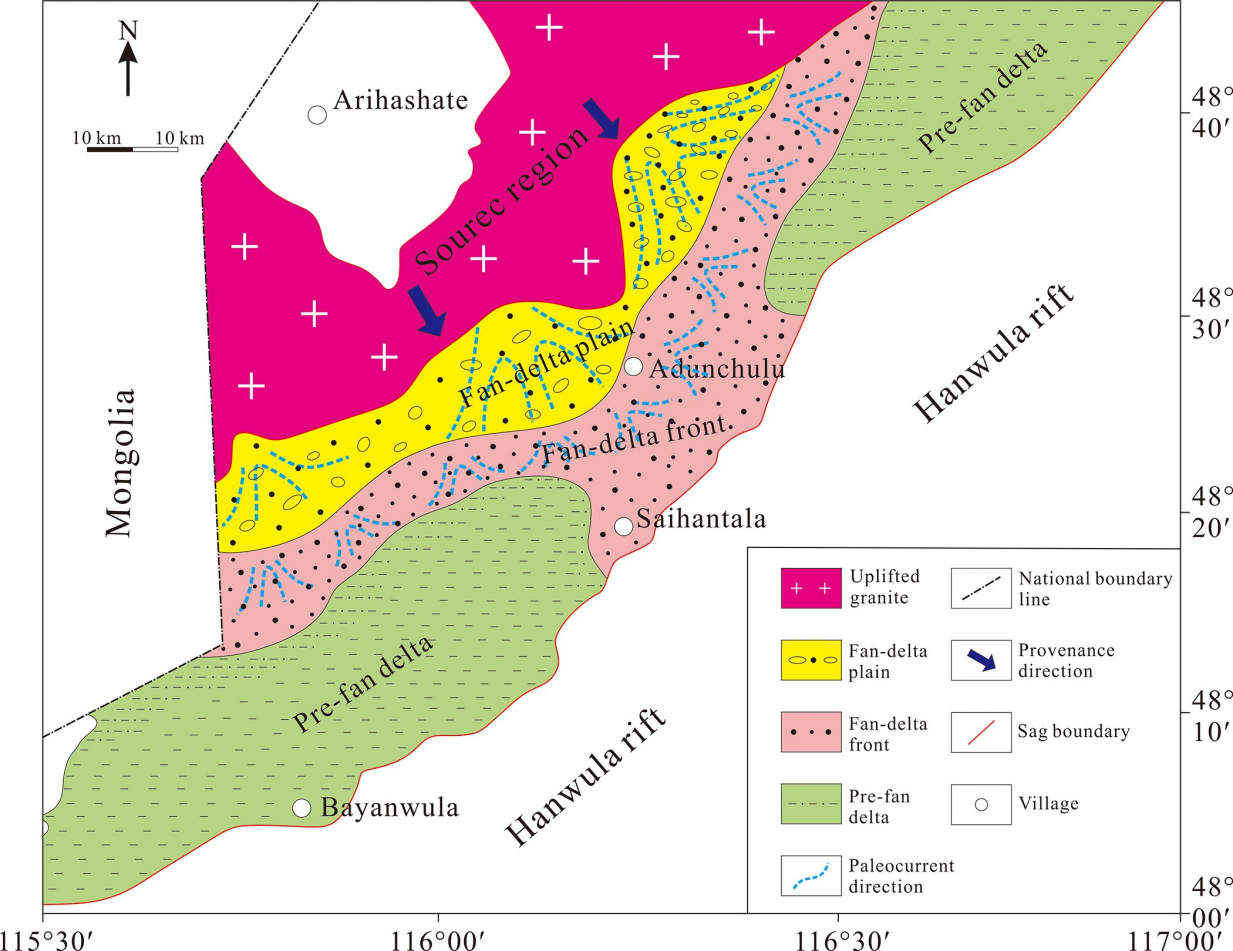
Sedimentary facies map of the Yimin Formation in the Kelulun Depression [19]. Republished from [Zhou W.B, Zhang R, Liu T, Mu H.Q, Zhao Z.W, Huang X. Metallogenic Geological Features and Prospective Study for Sandstone-type Uranium Deposit in Bel-Ulson Depression 2020; 53(03), 201–209.] under a CC BY license, with permission from [Northwestern Geology], original copyright [2020].

The deposition of the Yimin Formation occurred in the tectonic transition between rifting and subsidence, which shrank lake basins and formed a sedimentary system consisting of fan-delta plains, fan-delta fronts, and pre-fan deltas (Fig 17) [71,72]. The Adunchulu Uplift provided abundant matter to this steep monocline, promoting the formation of fan deltas inside the depression. Furthermore, due to the steep terrain, weathering and erosion on the western side of the source region created vast quantities of detrital matter, which were washed by floods into the basin and deposited in saddle landforms. Large near-source fan deltas filled with coarse clastic and sandy conglomerates were thus formed.

After the Yimin Formation was deposited, the terrain gradually became flattened, which decreased the difference in height between the depression and its surrounding uplifts. Consequently, the base of the alluvial fans and middle-fan subfacies on the western side were denuded, and the landscape inside the depression became dominated by fan-delta plains and fronts. The fan-delta plains spread along the depression in the NE direction. Their sediments are coarse and compositionally immature clastic and sandy conglomerates dominated by granite and brown-yellow or brick-red colors. Fan-delta fronts developed in the central and western parts of the depression, and their sediments are grey-colored medium- and fine-grained sandstones, with predominantly quartz and feldspar clasts. Organic clasts like detrital coal are also common. The most common facies here are underwater distributary channel, interdistributary bay, and estuary bar microfacies. Pre-fan deltas developed on the southern and northern ends of the depression, and their sediments are grey and dark-grey mudstones and siltstones intercalated with large quantities of detrital coal [73]. In summary, the sedimentary facies and sandbody distribution indicate that the materials of the Yimin Formation were derived from the Adunchulu Uplift at the western side of the Kelulun Depression.

#### 5.3.2 Constraints on provenance from zircon U-Pb ages

Age dating of geological bodies around the Kelulun Depression, i.e., the Adunchulu Uplift, Bayang Shan, and Erentaoleigai, revealed the spatiotemporal distribution of material sources around the Kelulun Depression (Fig 1c). The Hailar Basin has undergone many periods of granitic magmatism throughout its geological evolution, which formed acidic igneous rocks of varying ages in the southern part of Manzhouli [74,75]. Permian granites (280–335 Ma) mainly outcrop in the eastern part of the depression, around Erentaoleigai [76]. Most of the Middle-Late Triassic alkali feldspar granites (205–260 Ma) outcrop at the western part of the depression [77,78], at the Adunchulu Uplift. A few outcrops have also been observed at Chagantaoleigai, Bayan Shan, Jiawula-Chaganbulagen, and Erentaoleigai. Early Jurassic monzogranites and granite porphyries (175–185 Ma) mainly outcrop in the northern part of the depression [78,79], at Wunugetushan and Dashimo, whereas Late Jurassic–Early Cretaceous biotite granites and granite porphyries (125–160 Ma) are mainly located at the Adunchulu Uplift [80,81]. Mesozoic volcanism in southern Manzhouli started in the Middle-Late Jurassic and persisted until the Early Cretaceous, and its effects are most pronounced on the two sides of the Mongol-Okhotsk suture. Rhyolites, andesites, and olivine basalts with ages of 120–165 Ma were formed by this volcanism [82], and they mainly outcrop in the southern and northern parts of the depression (the Hanwula Uplift and Bayang Shan, respectively).

**Fig 18:**
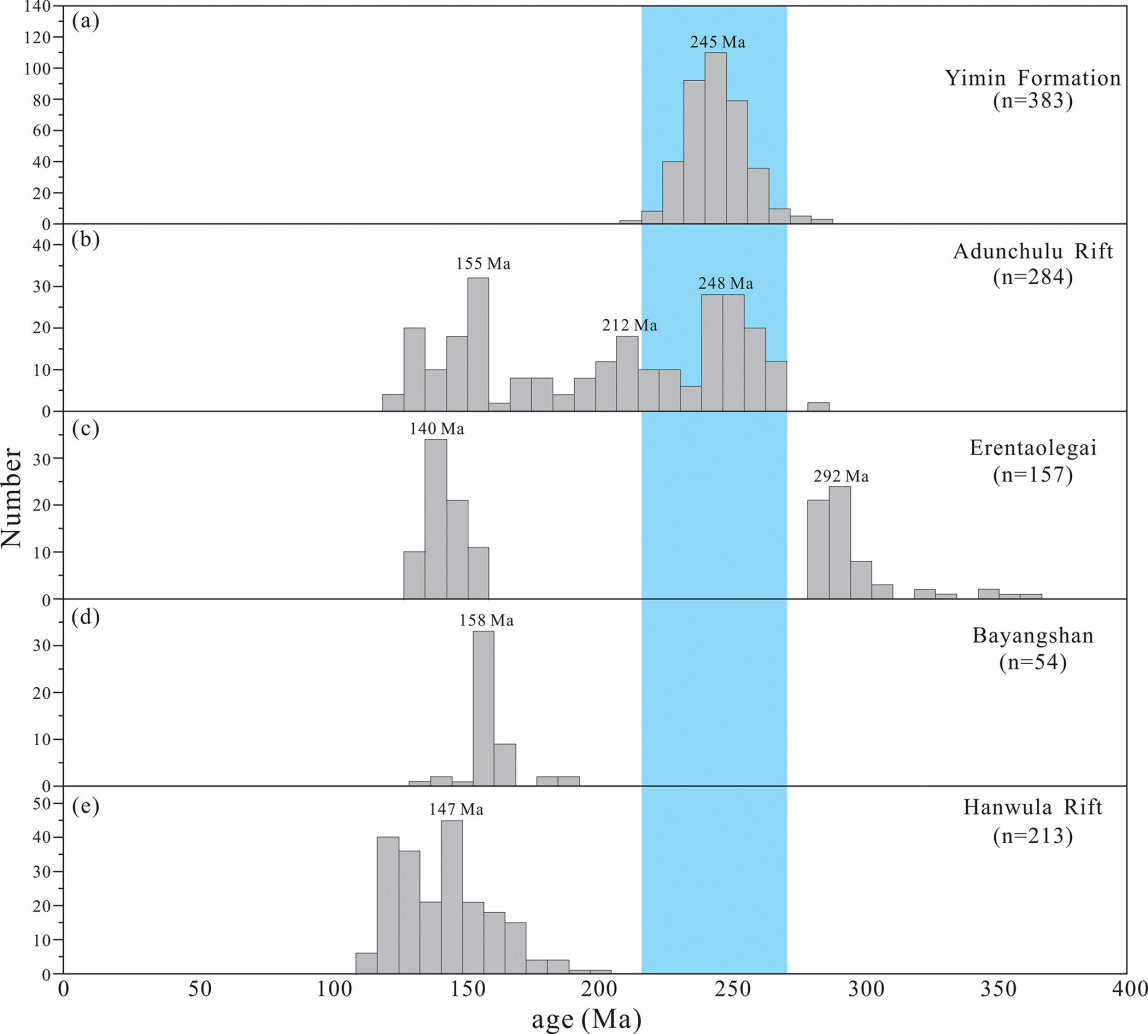
U-Pb age spectra of detrital zircons from the Yimin Formation sandstones and geological bodies around the Kelulun Depression [76–85]. (n represents the number of zircons).

The ages of the detrital zircons found in Yimin Formation sandstones range from 215 Ma to 287 Ma and are most commonly distributed in the range of 230–260 Ma. Therefore, the geological ages of the sources of these sandstones correspond to the Early-Middle Triassic and are consistent with the age peak of Triassic granites in the Adunchulu Uplift (western part of the depression) (Fig 18). In view of the lithologic, geochemical, and chronological evidence, we conclude that the detrital matter in the Yimin Formation was largely derived from Triassic granites in the Adunchulu Uplift. During the Triassic, large quantities of granite formed in southern Manzhouli due to the southward subduction of the Mongol-Okhotsk oceanic plate [83–85]. These granites subsequently underwent uplifting and denudation since the Late Cretaceous, which supplied ample quantities of detrital matter to the Kelulun Depression. Sourcing of Triassic granites related to an old subduction zone explains the ACM geochemical characteristics of the studied sandstones.

### 5.4 Source of uranium

Sandstone uranium deposits are derived from uranium-rich rocks and sedimentary layers in the erosion source-area as well as the ore-bearing stratum itself [86]. The scale of uranium mineralization is in part determined by the uranium content and outcropping area of the parent rock. If the ore-bearing strata receive large quantities of detrital uranium during sedimentation and diagenesis, their capacity to act as a source of uranium will be greatly enhanced. This also establishes a foundation for large-scale uranium mineralization [87].

In the Kelulun Depression, Late Paleozoic granites have uranium contents of 2.7 ppm to 22.3 ppm, while Mesozoic granites have uranium contents of 2.8 ppm to 5.4 ppm [88]. These uranium levels far exceed the average of the upper continental crust (2.80 ppm), indicating that the area is a good source [34]. Late Paleozoic granites in this area have Th/U ratios of 3.79–9.29 (6.33 on average) and uranium mobilization rates of 65%–75%, whereas Mesozoic granites have Th/U ratios of 4.23–8.16 (5.24 on average) and uranium mobilization rates of 55%–90%. These (high) Th/U ratios and uranium mobilization rates indicate that vast amounts of uranium may have migrated out of these rocks [89,90]. The uranium contents of the Yimin Formation strata range from 1.48 ppm to 5.50 ppm [71], and these estimates are higher than the global average for sandstones (0.45 ppm). Therefore, uranium-containing clasts may have migrated with subsurface waters into the depression during the weathering of uranium-rich granites in the erosion source area, which resulted in high uranium concentrations within the sandbody of the Yimin Formation. In addition, multiple rounds of fluid-induce dissolution during the diagenetic processes of the Yimin Formation created substantial secondary porosity. This increased the porosity of the sandbody and the interconnectivity of its pores, thus creating channels that facilitated the migration of uranium-containing oxygenated water. The dissolving fluids in the study area consist mainly of atmospheric precipitation and infiltrating waters, in the erosion source area, the bedrock fissure waters, porewaters, and groundwater have uranium concentrations of 14.4 ppm to 225 ppm, 130 ppm to 204 ppm, and 0.78 ppm to 243 ppm [13,15], respectively. Therefore, the atmospheric precipitation and infiltrating waters leached uranium from the parent rocks to create uranium- and oxygen-rich fluids, which migrated along the margins of the erosion source area into the Kelulun Depression. This created a stable and abundant source of uranium for the formation of sandstone uranium deposits in the Yimin Formation.

### 5.5 Constraints on uranium mineralization by the tectonic evolution of the source region

Beginning of rifting in the Late Jurassic: The Hailar Basin as a whole is a continental rift basin that has undergone multi-stage tectonic activities [60,91]. Before the Late Jurassic, the Hailar Basin was affected by the southward subduction of the Mongolia-Okhotsk Plate, resulting in extensive volcanic activity and the formation of a large number of Late Paleozoic granites in Manzhouli, southern Hailar Basin [92], which provided a rich material basis for the formation of mineralized strata and uranium source supply. Since the Late Jurassic, the upper crust was transformed from a compressional system to a tensional background due to the hot rise of the deep mantle, and the Hailar Basin was formed under the extensional background.

Pull-apart stage in the Early Cretaceous: Due to deep mantle upwelling, a change in the stress state of the upper crust from a compressional to extensional regime marked the beginning of an extensional pull-apart stage [91–93]. During this period, tectonic activity was extremely intense in the study area, resulting in vast differences in altitude between the basin and its surrounding uplifts. Many alluvial fans and fan-deltas formed as well as semi-deep and deep lake sediments, with only a few small sandbodies. These sandbodies did not undergo large-scale uplifting and denudation, preventing post-diagenetic oxidation from occurring on a large scale. These conditions are generally not conducive to the development of uranium deposits.

**Fig 19:**
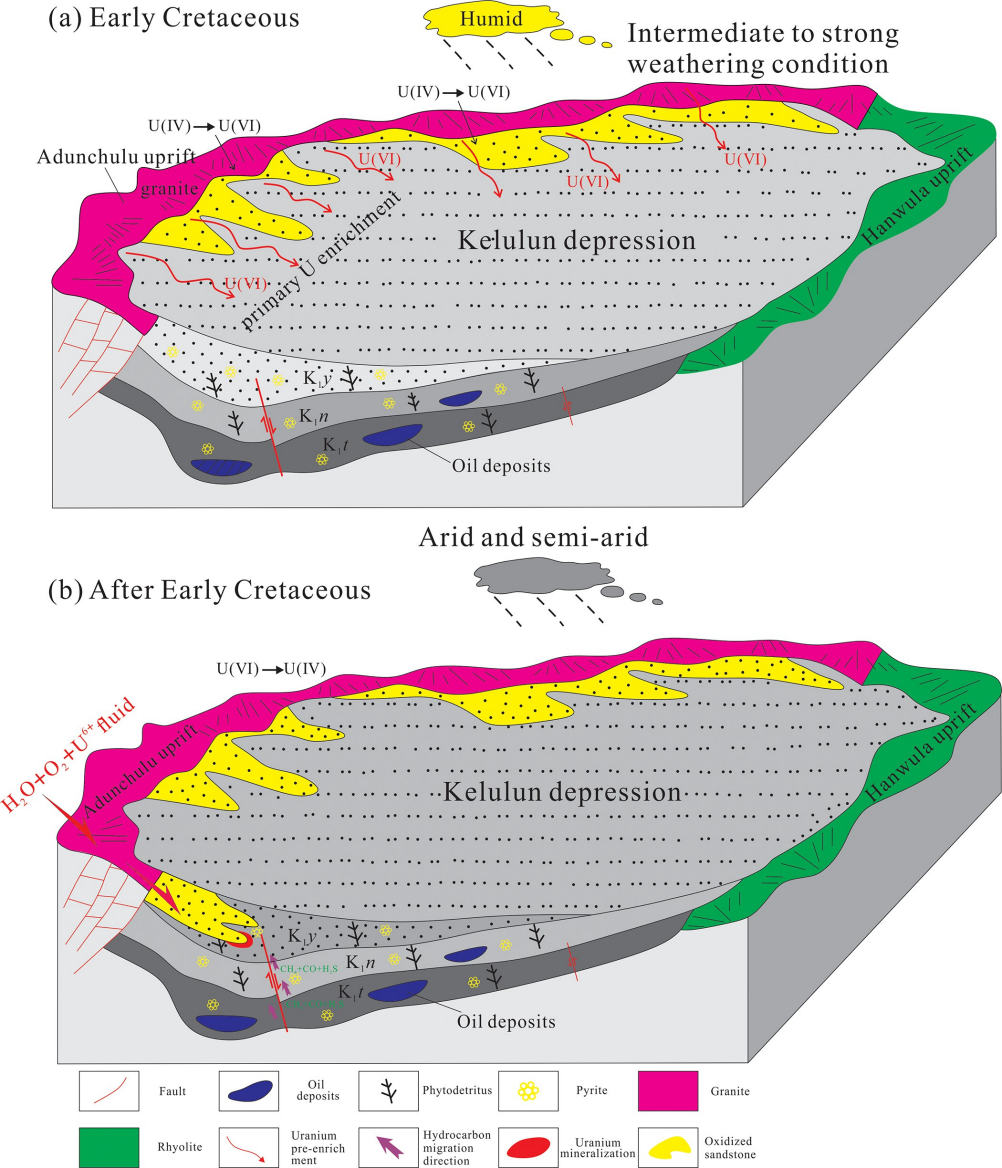
Stages of uranium mineralization in the sandstone uranium deposits of the Kelulun Depression [20].

Basin shrinkage in the middle-to-late Early Cretaceous: As the study area transitioned from rifting to subsidence, the rift basin also shrank, and the tectonic activity in the area started to subside [94–96]. The altitude disparity between the basin and its surrounding mountains started to decrease. In this stage, the Yimin Formation was largely comprised of grey-colored coal-bearing detrital matter. As such, its sandbody was loosely packed and large, with excellent lateral connectivity. Furthermore, it had a stable roof and floor made of impermeable shale. These conditions were conducive for the migration of oxygenated uranium-containing waters and the formation of uranium deposits. In addition, the prevailing paleoclimate during the deposition of the Yimin Formation was wet and warm, resulting in large quantities of plant detritus, detrital coal, and pyrite to mix with the grey sandbody [19,31]. Consequently, this sandbody has a high native reducing capacity and is also the main ore-bearing stratum in the study area. As the parent rocks had high uranium contents, the weathering of these rocks created large quantities of uranium-containing clasts, which then migrated into and deposited inside the depression. This effectively “pre-enriched” the strata of the Yimin Formation with relatively high levels of uranium (Fig 19A).

Tectonic inversion at the end of the Early Cretaceous–Late Cretaceous: At the beginning of this period, the extensional setting transformed into a compressional setting, and the sandstone uranium deposits began to form [97]. However, uplifting was less pronounced during this period, which limited the scale of uranium mineralization in the Yimin Formation. At the end of the Late Cretaceous, the Lower Cretaceous strata in the study area underwent compressional deformation, which caused large-scale uplifting and denudation in the Yimin Formation. This increased its hydraulic gradient and thus increased the recharge, runoff, and discharge of supergene fluids [98], which facilitated the infiltration of oxygenated uranium-containing waters into the Yimin Formation. As the Yimin Formation was also rich in reducing matter, such as detrital coal and pyrite, the U(Ⅵ) ions in the aforementioned fluid were reduced to U(Ⅳ) in its strata, which led to the formation of uranium orebodies in its redox transition zones (Fig 19B).

Basin shrinkage in the Paleogene–Neogene: The tectonic setting in the study area became stable, as the compressional stress became very weak [95,98]. The study area showed a tertiary planation surface, and the Yimin Formation was gradually modified by oxygenated uranium-containing waters, which increased uranium mineralization in its sandstone uranium deposits.

## 6. Conclusion

1. The Yimin Formation in the Kelulun Depression is mainly comprised of detrital sandstones with low compositional maturity. Based on the geochemical characteristics of these sandstones, the parent rocks are ACM felsic igneous rocks. The parent rocks have undergone a moderately high degree of weathering. The consequent denudation of these rocks provided an ample source of detrital matter in the study area.
2. The detrital zircon U-Pb ages of Yimin Formation sandstones range from 215 Ma to 287 Ma, with a peak at 230–260 Ma. Based on the zircon U-Pb ages, lithology, and geochemical characteristics of these sandstones, it was determined that the Yimin Formation matter was derived from Triassic granites in the Adunchulu Uplift, on the western side of the Kelulun Depression.
3. The Adunchulu Uplift is an outstanding source of uranium. Its uplift since the late Early Cretaceous and subsequent weathering and denudation of its uranium-rich granites played a crucial role in the formation of sandstone uranium deposits. The Kelulun Depression is a promising area for the exploration of sandstone uranium deposits.

## Supporting information

S1 Table Major element data of the Yimin Formation (%). Major element data. (XLSX)

S2 Table Trace and rare earth elements data of the Yimin Formation (ppm). Trace and rare earth elements data.
(XLSX)

S3 Table Detrital zircon U-Pb isotope data of the Yimin Formation sandstones. Detrital zircon U-Pb isotope data.
(XLSX)

S4 Table Detrital zircon trace element data of the Yimin Formation (ppm). Detrital zircon trace element data.
(XLSX)
